# Overview and Utilization of the NCI Thesaurus

**DOI:** 10.1002/cfg.445

**Published:** 2004-12

**Authors:** Gilberto Fragoso, Sherri de Coronado, Margaret Haber, Frank Hartel, Larry Wright

**Affiliations:** 1 NCI Center for Bioinformatics 6116 Executive Blvd Ste 403 National Cancer Institute, NIH Bethesda MD 20892 USA; 2 NCI Office of Communications 6116 Executive Blvd National Cancer Institute, NIH Bethesda MD 20892 USA

## Abstract

The NCI Thesaurus is a reference terminology covering areas of basic and clinical science, built with the goal of facilitating translational research in cancer. It contains nearly 110 000 terms in approximately 36000 concepts, partitioned in 20 subdomains, which include diseases, drugs, anatomy, genes, gene products, techniques, and biological processes, among others, all with a cancer-centric focus in content, and originally designed to support coding activities across the National Cancer Institute. Each concept represents a unit of meaning and contains a number of annotations, such as synonyms and preferred name, as well as annotations such as textual definitions and optional references to external authorities. In addition, concepts are modelled with description logic (DL) and defined by their relationships to other concepts; there are currently approximately 90 types of named relations declared in the terminology. The NCI Thesaurus is produced by the Enterprise Vocabulary Services project, a collaborative effort between the NCI Center for Bioinformatics and the NCI Office of Communications, and is part of the caCORE infrastructure stack (http://ncicb.nci.nih.gov/NCICB/core). It can be accessed programmatically through the open caBIO API and browsed via the web (http://nciterms.nci.nih.gov). A history of editing changes is also accessible through the API. In addition, the Thesaurus is available for download in various file formats, including OWL, the web ontology language, to facilitate its utilization by others.


**Comparative and Functional Genomics**
*Comp Funct Genom* 2004; **5**: 648–654.
Published online in Wiley InterScience (www.interscience.wiley.com). **DOI:** 10.1002/cfg.445

**Conference Paper**

### Overview and utilization of the NCI Thesaurus#

Gilberto Fragoso[1]*, Sherri de Coronado[1], Margaret Haber[2], Frank Hartel[1] and Larry Wright[2]
[1] *NCI Center for Bioinformatics, 6116 Executive Blvd, Ste 403, National Cancer Institute, NIH, Bethesda, MD 20892, USA*
[2] *NCI Office of Communications, 6116 Executive Blvd, National Cancer Institute, NIH, Bethesda, MD 20892, USA*

**Correspondence to:*
*Gilberto Fragoso, NCI Center for Bioinformatics, 6116 Executive Blvd, Ste 403, National Cancer Institute, NIH, Bethesda, MD 20892, USA.*
*E-mail: fragosog@mail.nih.gov*

*# This article is a U.S. Government work and is in the public domain in the U.S.A.*

Received: 21 November 2004
Accepted: 24 November 2004

### Abstract

**The NCI Thesaurus is a reference terminology covering areas of basic and clinical science, built with the goal of facilitating translational research in cancer. It contains nearly 110 000 terms in approximately 36 000 concepts, partitioned in 20 sub-domains, which include diseases, drugs, anatomy, genes, gene products, techniques, and biological processes, among others, all with a cancer-centric focus in content, and originally designed to support coding activities across the National Cancer Institute. Each concept represents a unit of meaning and contains a number of annotations, such as synonyms and preferred name, as well as annotations such as textual definitions and optional references to external authorities. In addition, concepts are modelled with description logic (DL) and defined by their relationships to other concepts; there are currently approximately 90 types of named relations declared in the terminology. The NCI Thesaurus is produced by the Enterprise Vocabulary Services project, a collaborative effort between the NCI Center for Bioinformatics and the NCI Office of Communications, and is part of the caCORE infrastructure stack (http://ncicb.nci.nih.gov/NCICB/core). It can be accessed programmatically through the open caBIO API and browsed via the web (http://nciterms.nci.nih.gov). A history of editing changes is also accessible through the API. In addition, the Thesaurus is available for download in various file formats, including OWL, the web ontology language, to facilitate its utilization by others. Published in 2005 by John Wiley & Sons, Ltd.**

**Keywords:** **biomedical terminology; ontology**

### Introduction

The NCI Thesaurus is a terminology developed and published by the Enterprise Vocabulary Services (EVS) project, a collaborative effort between the National Cancer Institute's (NCI) Office of Communications and the NCI Center for Bioinformatics (NCICB). It arose out of the need to have an institute-wide common terminology in order to integrate the diverse data systems in use throughout the NCI [10]. Coding of research-related activities helped to meet various responsibilities of the Institute; however, having codes assigned independently by various components of the NCI made it difficult to find and combine related information across programs. By incorporating the terms in use by the various programs and mapping them to unique concepts, it was intended that the NCI Thesaurus would facilitate interoperability and data sharing by the various components of the NCI. Its use within the institute has been expanded as part of the caCORE infrastructure being developed at the NCICB [5], where it is utilized as a source of base semantics and relations, in addition to its function as a source of codes for data annotation.

The NCI Thesaurus covers clinical and basic sciences as well as administrative areas. Although the content is cancer-centric, cancer research spans a broad area of biology, hence the Thesaurus can potentially be utilized outside the cancer research community. In this paper, we describe the NCI Thesaurus with the goal of helping researchers and organizations outside the NCI determine whether and how to utilize the Thesaurus. We offer an overview of the content areas in the terminology, including the various annotations with which the concepts are tagged, as well as the variety of roles that relate concepts in different domains. We also describe how two typical applications are utilizing the NCI Thesaurus.

**Table 1.** Counts of various entities in the NCI Thesaurus

| Thesaurus entity | Count |
|---|---|
| Kinds | 20 |
| Properties | 44 |
| Roles | 91 |
| Concepts | 36 364 |
| Defined concepts | 3960 |
| Terms | 107 672 |
| Concept w/definitions | 17 129 |

The contribution from retired concepts has not been taken into account. The value for Terms is taken from the FULL_SYN property. The value for concepts with definitions (last row) indicates the number of concepts with at least one definition; additional definitions per concept are not taken into account.

### Production cycle

The production cycle of the NCI Thesaurus is described in detail elsewhere [6]. Briefly, the development baseline of the Thesaurus is edited in the Terminology Development Environment suite of tools from Apelon Inc. (Ridgefield, CT). Each editor modifies his/her own database schema containing the terminology and periodically submits changesets to a workflow manager, who reconciles conflicts and generates and distributes new vocabulary baselines to each editor. The workflow process is based on the collaborative terminology development model (described in [2,3]). Editing history is processed in parallel when the baseline is slated for promotion to production [8]. Production baselines are published monthly in the Distributed Terminology Server (DTS), also from Apelon, and processed for distribution in Ontylog XML, the native format supported by the Apelon software, as well as in OWL [6,12] and flat file formats. Computer programs can access the Thesaurus in the DTS through the caBIO API [5]. Some metrics for the 04.11C (November, 2004) version of the vocabulary are shown in Tables 1 and 3.

### Structure of concepts

The basic unit of meaning in the NCI Thesaurus is the *concept*. A concept is denoted by a name and a code, and it must have a *kind* (see below for an explanation of kinds). Concepts are annotated with properties (name value pairs), and modelled in the Ontylog description logic (DL; see [7] for the semantics of Ontylog).

Roughly, properties in the Thesaurus are utilized to clarify concept meaning, either directly with information we generate/collect or indirectly through references to external authorities, and to support operations of dependent applications. In order to facilitate the correct coding of data in repositories, concepts are annotated with synonyms, textual definitions, references to external authorities, and various other pieces of information as deemed relevant for a particular domain. A full listing of the properties utilized in the Thesaurus can be found in **ftp://ftp1.nci.nih.gov/pub/cacore/EVS/ThesaurusSemantics/Properties.pdf**

The bulk of the content represented by properties in the Thesaurus involves terminology. All the terms associated with a concept are found in the FULL_SYN and Synonym properties. Both properties contain the same set of terms; however, the FULL_SYN contains additional information about the term, such as the term type and its source. One of the terms found in FULL_SYN is selected as the Preferred_Name and it is this term that is displayed by dependent applications to users; the Display_Name is utilized by applications when an alternative display term is required. Properties such as DEFINITION and DesignNote contain textual descriptions and additional clarifications on the recommended usage of the concept. When possible, references to external authorities are included, in properties such as Swiss-Prot and CAS_Registry (in the gene product and drug domains, respectively), as well as to NCI resources (e.g. NSC_Code). Many of the concepts also have references to UMLS CUIs (concept-unique identifiers assigned by the National Library of Medicine [11]), as mapped in the NCI MetaThesaurus (**http://ncimeta.nci.nih.gov**). Some properties are

**Table 2.** Roles instantiated in the disease domain of the Thesaurus in role expressions, showing the role names and the range kind of the roles

| Role name | Range kind |
|---|---|
| Disease_Excludes_Abnormal_Cell | Abnormal_Cell_Kind |
| Disease_Has_Abnormal_Cell | Abnormal_Cell_Kind |
| Disease_May_Have_Abnormal_Cell | Abnormal_Cell_Kind |
| Disease_Excludes_Normal_Cell_Origin | Anatomy_Kind |
| Disease_Excludes_Primary_Anatomic_Site | Anatomy_Kind |
| Disease_Has_Associated_Anatomic_Site | Anatomy_Kind |
| Disease_Has_Metastatic_Anatomic_Site | Anatomy_Kind |
| Disease_Has_Normal_Cell_Origin | Anatomy_Kind |
| Disease_Has_Normal_Tissue_Origin | Anatomy_Kind |
| Disease_Has_Primary_Anatomic_Site | Anatomy_Kind |
| Disease_May_Have_Normal_Cell_Origin | Anatomy_Kind |
| Disease_May_Have_Normal_Tissue_Origin | Anatomy_Kind |
| Disease_Excludes_Finding | Findings_and_Disorders_Kind |
| Disease_Has_Associated_Disease | Findings_and_Disorders_Kind |
| Disease_Has_Finding | Findings_and_Disorders_Kind |
| Disease_May_Have_Associated_Disease | Findings_and_Disorders_Kind |
| Disease_May_Have_Finding | Findings_and_Disorders_Kind |
| Disease_Excludes_Cytogenetic_Abnormality | Molecular_Abnormality_Kind |
| Disease_Excludes_Molecular_Abnormality | Molecular_Abnormality_Kind |
| Disease_Has_Cytogenetic_Abnormality | Molecular_Abnormality_Kind |
| Disease_Has_Molecular_Abnormality | Molecular_Abnormality_Kind |
| Disease_May_Have_Cytogenetic_Abnormality | Molecular_Abnormality_Kind |
| Disease_May_Have_Molecular_Abnormality | Molecular_Abnormality_Kind |
| Disease_Is_Grade | Properties_or_Attributes_Kind |
| Disease_is_Stage | Properties_or_Attributes_Kind |

**Table 3.** Breakdown of some metrics by domain in the Thesaurus

| Kind name | Concepts | Concepts with asserted roles | Roles asserted | Defined concepts |
|---|---|---|---|---|
| Abnormal_Cell_Kind | 553 | | | |
| Anatomy_Kind | 5079 | 2692 | 2805 | |
| Biological_Process_Kind | 1010 | 312 | 572 | |
| Chemicals_and_Drugs_Kind | 3589 | 246 | 1196 | |
| Chemotherapy_Regimen_Kind | 3168 | 3166 | 10 046 | |
| Clinical_or_Research_Activity_Kind | 1240 | | | |
| Diagnostic_and_Prognostic_Factors_Kind | 36 | | | |
| EO_Anatomy_Kind | 302 | | | |
| EO_Findings_and_Disorders_Kind | 1079 | 230 | 274 | |
| Equipment_Kind | 100 | | | |
| Findings_and_Disorders_Kind | 10 417 | 2264 | 5856 | 909 |
| Gene_Kind | 1963 | 1909 | 10 497 | 1533 |
| Gene_Product_Kind | 2324 | 2073 | 13 522 | 1518 |
| Molecular_Abnormality_Kind | 574 | | | |
| NCI_Kind | 2729 | 237 | 237 | |
| Organism_Kind | 449 | | | |
| Pathway_Kind | 442 | | | |
| Properties_or_Attributes_Kind | 908 | | | |
| Retired_Kind | 2558 | | | |
| Technique_Kind | 402 | 15 | 19 | |
| Total (-Retired) | 36 364 | 13 144 | 45 024 | 3960 |

The values in the last row represent the total counts per category. The values in columns three and four (denoted by the 'Roles' term in the column header) represent the roles directly asserted on concepts by editors; role inheritance is not taken into account.

utilized to express relations between concepts that we cannot represent in roles (see below) — such as Gene_Encodes_Product — because of classification cycles, or because a proper representation in a role has not yet been decided on, or because the information is perhaps too volatile. Other properties are intended to be used internally by the EVS, support legacy applications not yet fully utilizing the Thesaurus, or mapping to vocabularies required by other NCI programs (e.g. Related_MedDRA_Code).

As mentioned above, the terminology is being developed in a DL environment, so concepts are compositionally defined by their placement in a subsumption hierarchy and the role expressions that are asserted on each. DL is utilized in an effort to make the classification hierarchies in the terminology more consistent and information retrieval more accurate. Not all domains have been fully modelled in DL as of this writing, and some domains will have little, if any, modelling, as they are considered reference domains that the main content areas are modelled against. Our initial consideration for selecting roles to model any given domain or kind in the terminology follows the criteria that roles can be reproducibly applied by independent domain experts, and can serve a useful purpose in the terminology [1]. In addition, we name them so their intent is understandable [1]. In this initial stage, we normally select the minimal number of roles that will allow for concepts to be declared as defined, and give preference to those roles that can be applied throughout the entire kind, not just to a sub-domain within the kind. However, users of the Thesaurus frequently provide use cases for additional role relations in the terminology, and sub-domain independence ceases to be a consideration; the method we have adopted to help us extend the terminology in such instances is described elsewhere [7].

Role assertions provide detailed semantic relationships between domains, e.g. between neoplastic diseases and genes, that can be leveraged by dependent applications without resorting to DL reasoners. However, we cannot view role expressions as unconstrained semantic relationships to be utilized by users to find related information about an entity, as this can be in conflict with the formal utilization of roles in the DL. The support of non-defining associations by Ontylog in the near future will allow us to include this type of information (i.e. annotation of a concept with another concept) and support a wider range of use cases. As an illustration of their nature and scope, the roles instantiated in the disease domain are shown in Table 2; a full listing of the roles currently declared in the NCI Thesaurus is available online from **ftp://ftp1.nci.nih.gov/pub/cacore/EVS/ThesaurusSemantics/Roles.pdf**. Table 2 shows the type of information that is being captured via role expressions for concepts in the disease domain. It includes cell types, primary and metastatic anatomical sites, findings, associated diseases, and various types of molecular and cellular abnormalities. Some roles are considered definitional for a disease, others are not, and care is taken that all assertions are valid for all sub-types.

A summary of the extent of modelling per kind, as gauged by the number of concepts with directly asserted role expressions, is shown in Table 3. Nearly 35% of the concepts have directly asserted role relations, and many more inherit such relationships through the classification hierarchy. In the main modelling areas in the Thesaurus, this value ranges from 20% in the diseases and disorder kind to nearly 90–95% in the genes and gene product kinds. Still, the concepts modelled to the degree that they can be declared defined is roughly 10%, all in the gene, gene products and disease domains.

### Content areas

The NCI Thesaurus contains the terminology in use by the various divisions and offices of the NCI. Its coverage spans administrative, scientific and clinical areas, although because of its focus on cancer, it does not cover each domain exhaustively. In brief, the areas are encoded as *kinds* in the Thesaurus, which can be thought of as types or disjoint classes. Each concept can have only one kind, although concepts can be placed in multiple locations in the taxonomic trees describing that kind. The kinds are listed in Table 3; the full listing along with the kind definitions can be found in **ftp://ftp1.nci.nih.gov/pub/cacore/EVS/ThesaurusSemantics/KindDefinitions.pdf**.

A number of the kinds consist of trees with several upper level branches built on different organizing principles; while one branch is meant to be populated with concepts according to that branch's organizing principle, the rest of the branches are meant to serve as a reference that the main branch concepts can be modelled against (e.g. the *Protein, Organized by Structure* subheading of *Protein*). However, in some cases the ancillary branches contain concepts that cannot easily be placed under the main branch and which are better described by the alternative view these hierarchies provide. The non-administrative domain areas in the Thesaurus include genes, gene products, pathways, biological processes, anatomy, disorders, drugs and combination chemotherapies (Table 3). These might be of general interest for either the content or the model. Other, perhaps more specific areas, include molecular abnormalities, abnormal cell types, anatomy and disorders of experimental organisms, techniques and equipment.

The combination chemotherapy area merits mention as a unique, comprehensive resource. Cancer therapy is largely based on combinations of multiple drugs, and the NCI Thesaurus listing includes all combinations known to be used in either clinical trials or standard clinical practice. This domain is largely unstructured in the Thesaurus, and all the concepts in the domain are primitive (i.e. not defined in the DL sense), although almost all of them (about 99%) are related to their component drugs by role expressions.

The chemical and drug area is subdivided into structural and functional classification branches. The functional branch contains a number of subareas, such as *Pharmacologic Substance, Chemical Modifier, Reagent* and *Industrial Product*. The vast majority of concepts in this domain are primitive, and most are organized under *Pharmacologic Substance*, reflecting an interest in the utilization of drugs for cancer treatment. A great deal of work in this kind was initially done in a separate experimental database, with the goal of creating a drug model that can be shared with other US federal agencies and organizations, including the Veterans Health Administration, the Food and Drug Administration, and the National Library of Medicine. Integration and fleshing out of the drug model in the production version of the Thesaurus is currently ongoing, and will relate drugs to molecular entities such as proteins, to biological processes and physiologic effects, and to diseases. Additional work on terminology in the nutrient and chemoprevention areas is also under way.

The anatomy domain is one of the most general in the Thesaurus. It is partitioned into several sub-branches spanning gross anatomy through microanatomy, and includes fluids, cavities and entities at the molecular level. Constituent parts of anatomical sites are placed in distinct shallow branches built with the is_a relation and related through part_of-type role expressions to the anatomical whole. Although has_location roles have been instantiated, the vast majority of relations in the present version of the anatomy kind involve the part_of role. The resulting graph provides a view of the domain that is considered very useful as a reference kind to model diseases against. It has been reviewed and recommended for coding purposes by the Consolidated Health Informatics group [4]. We do not envision major changes in this domain; however, ongoing analysis in collaboration with the Jackson Laboratory and other interested parties is expected to result in a harmonization of this domain with other terminologies, including those for mice and other experimental organisms.

The findings and disorders kind contains more than 10 000 concepts, including 6500 neoplastic as well as many non-neoplastic disease concepts of special interest to the Institute. The top concept for the neoplastic diseases is *Neoplasm*, and most of the concepts in this domain are doubly treed under both *Neoplasm by Morphology* and *Neoplasm by Site* subheadings. All neoplasm concepts have at least some role modelling, with an average of slightly over 10 roles per concept, largely based on characteristics inherited through an extensive, multi-faceted classification hierarchy. Nevertheless, most of the concepts are still primitive in the DL sense; modelling in this area is proceeding at a fast pace, with a large effort devoted to the proper expression of relationships between concepts both within and outside the kind (see above and Table 2). Partly because of the increasing focus on molecular signatures of diseases, concepts in this domain require the largest number of role expressions before they can be considered defined. Further, and as mentioned elsewhere, some non-defining relations convey information considered useful by clinicians. Because of the terminology content, as well as the semantic relations, the disease domain is one of the richest areas in the Thesaurus.

The gene kind contains cancer-related genes organized according to biological function. In addition to oncogenes and tumour suppressors, this kind includes genes that are not known to be involved in oncogenesis; however, they have been added because, for instance, they might participate in processes related in some fashion to oncogenesis, e.g. cell cycle. This kind is difficult to model because the main defining characteristic of a gene, its sequence, does not belong in a terminology. Nevertheless, the set of relations required to declare a gene concept defined, which includes pathway, process and disease associations, can distinguish between the vast majority of cases; chromosomal location is usually the only feature that can be used to distinguish between members of gene families. Some restructuring of the domain is still deemed necessary, however.

In terms of content, the gene product kind is very similar to the gene kind. It contains protein and RNA products that are either directly related to, or have some type of association with, oncogenesis. The concepts are organized by function, but the domain also contains trees that describe alternative organizations by location and structure. Because of the active role of gene products in normal and abnormal processes, concepts in this domain are related to a wider number of reference domains than their reference genes.

The biochemical pathways domain is considered a reference kind and has been modelled minimally. It consists of named pathway concepts derived from KEGG [9] and BioCarta (**http://www.biocarta.com/**), organized in a simple shallow hierarchy of general concepts, such as *Metabolic Pathway, Regulatory Pathway* and *Signaling Pathway*. No roles have been declared in this domain; however, this kind is the range for roles declared in the gene and gene product domains, e.g. Gene_Product_Is_Element_in_Pathway. Although this area is of great importance, and can serve to define genes and gene products, it was not included in the Thesaurus until recently because of issues related to naming conventions and the start and endpoints of pathways. We anticipate some restructuring as the field advances.

The biological process domain covers the range from *Subcellular Process* through *Population Process*. These disjoint biological areas are spanned by *Pathologic Process*. Although broad, the hierarchies are not very deep. Our main interest in this area are the pathological processes, although normal biological processes must be represented to a certain extent — in some instances for their terminology, in other instances as a source of role values used to define concepts in other domains.

### Utilization of the NCI Thesaurus

As mentioned above, the main use for the Thesaurus is to provide codes for annotation of artifacts in NCI data repositories. Because it is being developed in DL, dependent applications are also beginning to utilize its semantic relationships to allow users to navigate between domains. As it can be accessed freely, the EVS does not collect information on its use; however, two examples of applications utilizing the Thesaurus in the NCICB are caIMAGE (**http://cancerimages.nci.nih.gov/**) and the caDSR (**http://ncicb.nci.nih.gov/core/caDSR**).

The caIMAGE (cancer Images database) is a portal application supporting users of the Mouse Models of Human Cancers Consortium (MMHCC). Data is coded with anatomical locations as well as with diseases. When submitting or searching for specific images, users are presented with a tree browser of the anatomy domain, so that they can select the most specific concept that represents the anatomical location of the image. Once a specific anatomical system is selected, a second tree browser is displayed that selects a branch in the diseases domain with a relation to the anatomical site already entered. The branch selected for display is the most specific that can be returned based on the relations asserted between domains; if no relation to a disease has been asserted for a specific anatomical concept, the application walks up the hierarchy and selects the next concept it finds with the appropriate relation asserted. This application helps users to select annotations with the highest degree of specificity.

The caDSR (cancer Data Standards Repository) is an ISO 11 179 metadata registry originally developed to help store and maintain common data elements utilized in case report forms. Vocabulary for the metadata is now drawn exclusively from the vocabularies available through the EVS, mainly the Thesaurus. The administrative and curation tools enforce this dependency; if no suitable term is found by a curator in the Thesaurus when creating a data element, for instance, the user is required to request assistance from EVS editors which, if necessary, will create a new concept and return its code, preferred name and a definition.

### Future prospects

The NCI Thesaurus is not a finished product and will continue to evolve, hopefully gracefully. Although it contains terminology already in use in the NCI, new applications are being developed that require very often not only the inclusion of additional concepts and terms, but a specific set of relations between concepts. In addition, some changes might be more significant than others. When developing a new domain area in the Thesaurus (five within the last 12 months), it is not always clear what is the best structure and organization to satisfy the user requirements; we err on the side of caution by maintaining these areas in their own kinds while use cases are refined and our own integration concerns are addressed. This is an operational issue; we need to begin serving new terminology very quickly, yet maintain the flexibility to integrate a new area within the existing structure(s) of the Thesaurus — our experience has been that it is easier to integrate kinds than it is to split one into two disjoint areas. As terminology evolution is to be expected, concept history tracking and querying mechanisms should be considered a necessity.

Some areas in the Thesaurus are less specific to cancer than others. They provide some basic terms and concepts, and serve to model against; however, they still require resources to maintain and enhance them. We recognize that the ability to integrate other terminologies into the Thesaurus in a way that would respect the source's integrity, as well as our own operational and editorial constraints, would allow us to concentrate more on the main cancer-related content of the Thesaurus. This concern is not restricted to us. A common language for vocabulary developers, such as OWL, which supports namespaces, offers prospects in this regard.

### References

1. Bakken S, Parker J, Konicek D, Campbell KE. 2000. An evaluation of ICNP intervention axes as terminology model components. *Proceedings of the AMIA Symposium* 2000: 42–46.
2. Campbell KE, Cohn SP, Chute CG, Rennels G, Shortliffe EH. 1996. Galapagos: computer-based support for evolution of a convergent medical terminology. *Proceedings of the AMIA Annual Fall Symposium 1996*: 269–273.
3. Campbell KE, Cohn SP, Chute CG, Shortliffe EH, Rennels G. 1998. Scalable methodologies for distributed development of logic-based convergent medical terminology. *Methods Inf Med* **37**(4–5): 426–439.
4. Consolidated Health Informatics, Standards Adoption Recommendation, Anatomy/Physiology. 2004. **http://www.whitehouse.gov/omb/egov/downloads/aandp_full_public.doc**.
5. Covitz PA, Hartel F, Schaefer C, *et al.* 2003. caCORE: a common infrastructure for cancer informatics. *Bioinformatics* **19**(18): 2404–2412.
6. Golbeck J, Fragoso G, Hartel F, Hendler J, Oberthaler J, Parsia B. 2003. The National Cancer Institute's Thesaurus and ontology. *J Web Semant* **1**(1): 75–80.
7. Hartel FW, de Coronado S, Dionne R, Fragoso G, Golbeck J. 2005. Modelling a description logic vocabulary for cancer research. *J Biomed Inform* (in press).
8. Hartel FW, Fragoso G, Ong KL, Dionne R. 2003. Enhancing quality of retrieval through concept edit history. *AMIA Annual Symposium Proceedings2003*: 279–283.
9. KEGG: Kyoto encyclopedia of genes and genomes; **http://www.genome.jp/kegg/**.
10. National Cancer Advisory Board, September 1997. **http://deainfo.nci.nih.gov/advisory/ncab/archive/minutes/103_0997/ncab0997.htm#7**.
11. Unified Medical Language System; **http://www.nlm.nih.gov/research/umls/**.
12. Web Ontology Language (OWL); **http://www.w3c.org/2004/OWL/**.
